# Prehospital survival of patients with ST-elevation myocardial infarction requiring out-of-hospital cardiopulmonary resuscitation - a nationwide, real-world observational study

**DOI:** 10.1186/s12873-025-01292-y

**Published:** 2025-07-18

**Authors:** Dominika Szabó, András Szabó, Andrea Székely

**Affiliations:** 1 Doctoral College, Semmelweis University, Üllői út. 26, Budapest, 1085 Hungary; 2 National Ambulance Service, Szauter F. u. 2/A, Győr, 9024 Hungary; 3 Department of Oxiology and Emergency Medicine, Semmelweis University, Üllői út 26, Budapest, 1085 Hungary; 4 Department of Anesthesiology and Intensive Therapy, Semmelweis University, Üllői út 78, Budapest, 1082 Hungary

**Keywords:** STEMI, OHCA, CPR, Prehospital survival, Initial rhythm

## Abstract

**Background:**

The mortality risk of patients presenting with ST-elevation myocardial infarction (STEMI) has been extensively researched. Even though STEMI can be diagnosed before hospital admission, prehospital mortality has been less frequently studied. We aimed to analyze the outcomes of patients with STEMI requiring out-of-hospital cardiopulmonary resuscitation (CPR).

**Methods:**

From a large, nationwide prehospital case report database, we collected data from 668 patients requiring CPR because of ambulance-witnessed OHCA (out-of-hospital cardiac arrest) who were diagnosed with STEMI by ECG before cardiac arrest. Utstein-style consensus reporting guidelines were followed. The endpoint was hospital admission with spontaneous circulation. In addition to descriptive statistics, we also aimed to identify predictors of the outcome using multivariable logistic regression. Model performance was characterized by c-statistics and multiple fitting methods. Internal validation was performed using calibration intercept and slope.

**Results:**

Using CPR initial rhythm, age, initial heart rate, initial systolic blood pressure, and ECG localization of STEMI as predictors, we found that the constructed logistic regression model showed good discriminative ability, with a c-statistic of 0.844 (95% CI = 0.8105–0.8787). The overall model fit was good, with Hosmer & Lemeshow *p* = 0.979. The value of Nagelkerke R^2^ test of 0.445 indicated a strong relationship between predictors and outcome. The Z-value of calibration slope was relative to slope = 1 (95% CI = 0.85–1.16).

**Conclusions:**

This model can be used to estimate the probability of hospital admission following resuscitation due to ambulance-witnessed OHCA in patients with STEMI. Further studies are needed to improve the possibility of definitive in-hospital treatment for a better survival rate.

**Clinical trial number:**

Not applicable.

## Background

The mortality risk of patients presenting with ST-elevation myocardial infarction (STEMI) has been extensively studied. Several scoring algorithms have been developed and validated to assess individual risk [1]. These studies included only data from patients who were successfully admitted to the hospital, even though STEMI can be diagnosed before hospital admission. Prehospital mortality of patients with STEMI has been less frequently studied: in fact, these patients have not even been analyzed separately in the largest out-of-hospital cardiac arrest (OHCA) registries [2, 3]. 

We have previously shown that the probability of prehospital death following CPR (cardiopulmonary resuscitation) due to OHCA in patients with STEMI may be estimated by age, initial rhythm, and the presence or absence of spontaneous circulation at ambulance arrival. Patients who have spontaneous circulation when the emergency service arrives but subsequently develop cardiac arrest due to a non-shockable rhythm may be at extremely high risk of out-of-hospital death [4]. 

Despite the fact that STEMI can be diagnosed before hospital admission, clinical trials and registries almost exclusively begin after hospital admission. Coronary intervention and modern medical treatment have improved outcomes. In order to achieve the highest proportion for definitive treatment possible, we need to pay attention to patients who have been diagnosed with STEMI on the basis of symptoms and ECG, but who died at the scene or during transport, before hospital admission.

The structured, quality-controlled, digital case report system of the Hungarian National Ambulance Service offers the opportunity to analyze the incidence, clinical features, and prehospital outcomes of patients, who were diagnosed with STEMI in a prehospital setting and suffered ambulance-witnessed cardiac arrest.

After the characterization of the highest-risk groups, further studies are needed to compare prehospital diagnostic and therapeutic alternatives for these patients to improve the possibility of definitive in-hospital treatment to improve survival.

## Methods

### The aim of the study

In this study, we aimed to analyze the incidence, clinical features and prehospital outcomes of patients requiring out-of-hospital CPR following the diagnosis of STEMI with 12-lead ECG (electrocardiogram) using the nationwide database of structured case reports of the National Ambulance Service in Hungary.

### Design and setting of the study

The anonymized database was queried using electronic case report software (ESETLAP), which is used to write digital, structured, and quality-controlled case reports by the advanced ambulance units of the National Ambulance Service of Hungary. From November 2018 until July 2024, we collected data from 668 patients requiring CPR for OHCA who were diagnosed with STEMI using ECG before cardiac arrest. We screened and included patients who met the following criteria:

- complaints and symptoms of myocardial ischemia were reported at the time of the emergency call;
- symptoms and diagnosis were confirmed in the case report and/or registered using International Classification of Diseases (ICD) codes;
- a 12-lead ECG test was performed before cardiac arrest;
- new ST-segment elevations in at least two contiguous leads or new bundle branch blocks with ischemic repolarization patterns [5, 6] were registered using the structured digital case report system;
- cardiac arrest was witnessed by the ambulance, and.
- the documentation is clear and complete.

In order to reduce noise due to the questionable diagnostic specificity of post-RoSC (return of spontaneous circulation) ST-deviations and novel bundle branch blocks [7], we did not include the STEMI cases diagnosed after RoSC in this analysis.

Patients who complained of symptoms that lasted longer than 24 h or who had atypical symptoms (e.g., chest pain associated with limb movement or breathing or documented suspicious signs of pulmonary embolism) were excluded.

The outcome measure was hospital admission with spontaneous circulation.

Data from 39 patients who received ongoing CPR upon arrival at the hospital were excluded from the final analysis due to lack of knowledge of patient outcomes and in order to compare binary outcomes.

### Outcome and statistical analysis

The available baseline demographic and clinical characteristics are listed in the tables. Medians and interquartile ranges were calculated for continuous variables. Parametric values are listed as counts and frequencies (percentages). The baseline characteristics of patients were statistically compared between survivors (successful CPR, alive at hospital admission, *n* = 237) and non-survivors (unsuccessful CPR, *n* = 392), excluding patients transferred/admitted with continuous CPR (*n* = 39). Nonparametric values were analyzed using the Mann-Whitney U Test, and parametric values were compared using the chi-squared test. A two-tailed p value less than 0.05 was considered to indicate statistical significance.

In addition to descriptive statistics, we aimed to identify patient factors associated with outcome measures using multivariable logistic regression based on cases with nonmissing data (*n* = 579), also considering the overall AIC value, with lower values indicating better fit. Model discrimination performance was characterized by c-statistics. Nagelkerke R^2^ and Hosmer-Lemeshow test were also calculated to estimate model fit. Internal validation was performed using calibration intercept and slope.

The Utstein-style consensus reporting guidelines [8] and the TRIPOD checklist [9] were used for data collection, analysis, and interpretation processes.

The use of anonymized data for this study was approved by the head of the institution and the Regional Ethics Committee on Human Research of Semmelweis University (approval number: SE RKEB: 238/2024). This study was conducted according to the principles of the Declaration of Helsinki.

The database query was originally performed using Microsoft Office 365 Excel version 2503 (Microsoft Corp, Redmond, Washington). Statistical analysis and graphical interpretation were performed using IBM SPSS Statistics for Windows, Version 25.0. (IBM Corp., Armonk, NY, 2017), jamovi 2.6. (The jamovi project (2024) Sydney, Australia), furthermore pROC and pmcalibration packages in R version 4.4.2. (R Foundation for Statistical Computing, Vienna, Austria, 2024) [10, 11]. 

## Results

### Demographics and clinical presentation

Among the 629 cases, 237 (37.7%) survived until hospital admission, 392 patients (62.3%) died at the scene. The available baseline demographic and clinical data are summarized in Table 1.

**Table 1:** Descriptive statistical analysis of available baseline data

Baseline characteristics		Total		RoSC, hospital admission		Unsuccessful, dead at the scene		
		*n* = 629		*n* = 392		*n* = 237		
**Nonparametric variables**		**Valid ** ***n***	**%**	**Median**	**IQR**	**Median**	**IQR**	**Mann-Whitney ** ***p***
Age (years)		625	99.4%	65	56–72	70	62–78	< 0.001
Systolic blood pressure (mmHg)		582	92.5%	135	110–155	114	85–144	< 0.001
Heart rate (1/min)		618	98.3%	85	70–100	90	68–111	0.031
First etCO2 (mmHg)		315	50.1%	28	20–38	14	10-22.5	< 0.001
**Categorical variables**		***n***	**Total ** ***n *** **%**	***n***	**Row *n *** **%**	**n**	**Row *n *** **%**	**Chi-squared p**
Sex	Male	375	59.6%	240	64.0%	135	36.0%	0.266
	Female	350	39.7%	149	59.6%	101	40.4%	
	missing	4	0.6%	3	75.0%	1	25.0%	
Consciousness	alert	423	67.2%	301	71.2%	122	28.8%	< 0.001
	disturbed consciousness	206	32.8%	91	44.2%	115	55.8%	
CPR initial rhythm	non-shockable	290	46.1%	90	31.0%	200	69.0%	< 0.001
	shockable	339	53.9%	302	89.1%	37	10.9%	
Defibrillation	not required	246	39.1%	81	32.9%	165	67.1%	< 0.001
	required	383	60.9%	311	81.2%	72	18.8%	
ECG localization	anterior	262	41.7%	180	68.7%	82	31.3%	0.001
	inferior	269	42.8%	168	62.5%	101	37.5%	
	lateral or other	32	5.1%	17	53.1%	15	46.9%	
	LBBB	29	4.6%	13	44.8%	16	55.2%	
	RBBB	37	5.9%	14	37.8%	23	62.2%	

Abbreviations: CPR: cardiopulmonary resuscitation, etCO2: end-tidal carbon dioxide, IQR: interquartile range, LBBB: left bundle branch block, min = minute, mmHg: millimeters of mercury, n = number RBBB: right bundle branch block, RoSC: return of spontaneous circulation

### Multivariable logistic regression algorithm: development and internal validation

The most important predictor for estimating hospital admission with spontaneous circulation was the initial rhythm of CPR. Shockable initial rhythm, as witnessed by the ambulance anticipated odds ratio of 18.14 (95% CI: 11.89–27.67, *p* < 0.001) for prehospital survival of patients with STEMI, compared to patients with non-shockable initial rhythm.

Additional variables were included in the model using forward selection based on likelihood ratio tests, total Akaike’s information criterion (AIC), Nagelkerke R^2^, c-statistics and Hosmer & Lemeshow goodness-of-fit test, with and without each variable, until well-fitting model was developed (see Tables 2 and 3).

**Fig. 1:**
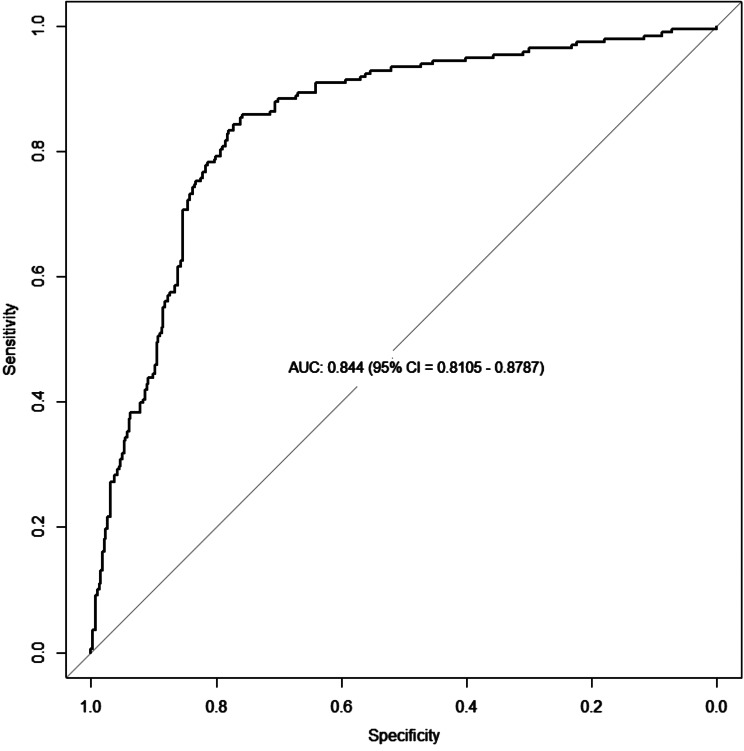
ROC analysis of the model in prediction of hospital admission with spontaneous circulation: the constructed logistic regression model showed excellent discriminative ability. Abbreviations: AUC: Area Under Curve, CI: Confidence Interval, ROC: Receiver Operating Characteristics

Using the CPR initial rhythm, age, initial heart rate, initial systolic blood pressure, and the ECG localization of STEMI as predictors, we found that the constructed logistic regression model showed excellent discriminative ability, with a c-statistic of 0.844 (95% CI = 0.8105–0.8787), as shown in Fig. 1.

**Fig. 2:**
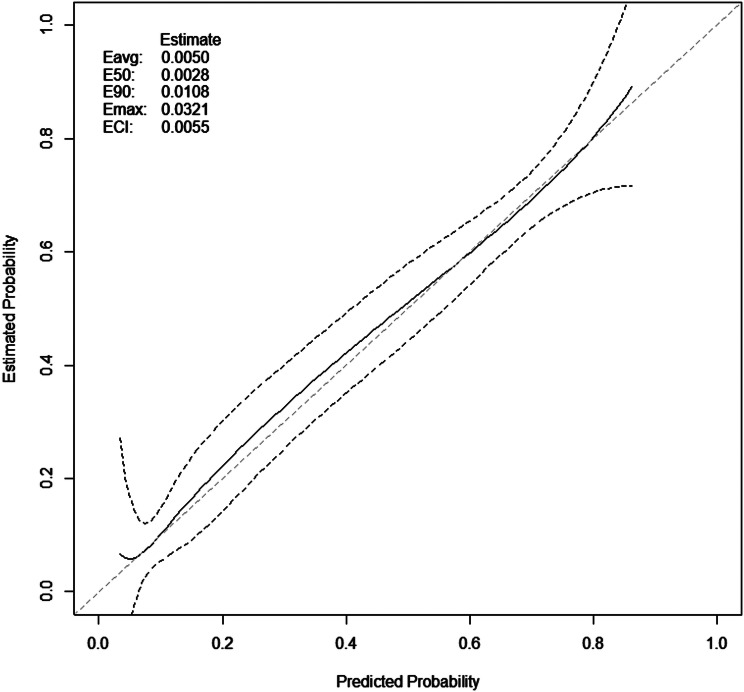
Calibration plot. Calibration metrics are based on a calibration curve estimated for a binary outcome using logit transformed predicted probabilities. Abbreviations: Eavg: Average absolute difference (integrated calibration index); E50: Median absolute difference; E90: 90th percentile absolute difference; Emax: Maximum absolute difference; ECI: Average squared difference (estimated calibration index)

The overall model fit was good, as Hosmer & Lemeshow *p* = 0.979 (chi-square: 2.055, df [degrees of freedom] = 8). The Nagelkerke R^2^ value of 0.445 indicated a strong relationship between the predictors and outcome. The graphical assessment of the calibration, and calibration metrics are presented in Fig. 2. and Table 4.

**Table 2:** Development of the logistic regression model

Model	Predictors	Likelihood ratio test			AIC	Nagelkerke R2	c-statistics	Hosmer & Lemeshow *p*
		chi-squared	df	*p*				
1.	Initial rhythm	240	1	< 0.001	597	0.433	0.807	-
2.	Initial rhythm	242	2	< 0.001	593	0.437	0.824	0.951
	Age							
3.	Initial rhythm	240	3	< 0.001	580	0.441	0.834	0.280
	Age							
	Heart rate							
4.	Initial rhythm	219	4	< 0.001	535	0.436	0.838	0.545
	Age							
	Heart rate							
	Systolic blood pressure							
5.	Initial rhythm	225	8	< 0.001	537	0.445	0.844	0.979
	Age							
	Heart rate							
	Systolic blood pressure							
	STEMI localization							

Forward selection was based on different statistical discrimination and calibration tests (see text). Abbreviations: AIC: Akaike’s Information Criterion, df: degrees of freedom

**Table 3:** Multivariate logistic regression analysis

Predictor	Estimate	95% Confidence interval		SE	Z	*p*
		Lower	Upper			
Intercept	3.34536	1.68468	5.00604	0.84730	3.948	< 0.001
Initial rhythm:						
Non-shockable– Shockable	-2.72419	-3.20067	-2.24772	0.24310	-11.206	< 0.001
Age (years)	-0.01405	-0.03156	0.00345	0.00893	-1.573	0.116
Heart rate (1/min)	-0.00839	-0.01606	-0.00072	0.00391	-2.145	0.032
Systolic blood pressure (mmHg)	0.00454	-0.00176	0.01084	0.00321	1.413	0.158
STEMI localisation:						
inferior– anterior	-0.05465	-0.54259	0.43329	0.24895	-0.220	0.826
lateral or other– anterior	-0.80509	-1.80715	0.19697	0.51127	-1.575	0.115
RBBB– anterior	-0.71799	-1.64503	0.20905	0.47299	-1.518	0.129
LBBB– anterior	0.32810	-0.59862	1.25482	0.47283	0.694	0.488

Multivariable logistic regression analysis was based on cases with nonmissing data (*n* = 579). The estimates show log odds of outcomes: RoSC, hospital admission vs. Unsuccessful, dead at the scene. Abbreviations: LBBB: left bundle branch block, min = minute, mmHg: millimeters of mercury, RBBB: right bundle branch block, SE: standard error

**Table 4:** Calibration intercept and slope

	Estimate	SE	z	Pr(>|z|)	Lower	Upper
Calibration Intercept	-1.2e-15	0.110	-1.1* e-14	1	-0.22	0.21
Calibration Slope	1.0e + 00	0.079	2.2e-14	1	0.85	1.16

Z-value for calibration slope is relative to slope = 1. Lower and upper values are the limits of 95% profile confidence intervals. Abbreviations: SE: standard error, Pr: prediction

### Limitations

The parameters collected are based on well-structured, and checked digital documentation written by ambulance teams; nevertheless, they may contain noise, as in other clinical studies. A higher quality and more detailed database could be achieved if the available data were recorded directly from patient monitoring devices.

The regions were not proportionally represented due to the gradual implementation of the digital, structured case report system of the National Ambulance Service during the study period. Traditional paper-based case reports were not analysed in this study.

Additionally, the database does not include data on patients who were not treated or transferred by any of the advanced life support (ALS) units of the Hungarian National Ambulance Service, such as patients who were resuscitated exclusively by an Air Ambulance Team, or by a local general practitioner alone; this may slightly affect the completeness of the collected data.

Since the study focused exclusively on the out-of-hospital setting, subsequent in-hospital decisions were not considered, as they should not influence prehospital emergency care. Therefore, further diagnostic and therapeutic decisions, such as confirming type 1 myocardial infarction or determining the need for a coronary intervention, were not analyzed.

Patients diagnosed with STEMI post-RoSC, and those without documented 12-lead ECGs were not included in this analysis based on the predefined inclusion criteria. Therefore, we had an uncertain number of patients who might have suffered out-of-hospital cardiac arrest due to acute coronary occlusion, but it was not possible to record an ECG beforehand.

Patients who underwent ongoing CPR upon hospital admission were excluded from the analysis due to medical, ethical and statistical considerations, given that further outcomes remain unknown. Their analysis would require a separate targeted study according to the hospital facilities.

## Discussion

### Main findings

Almost two-thirds of patients who were opinionated with STEMI in prehospital setting and suffered ambulance-witnessed cardiac arrest died out-of-hospital but narrowly missed being admitted to hospital and receiving definitive therapy. Our results show that the CPR initial rhythm, age, first measured heart rate and blood pressure, and the ECG localization of STEMI can be used to estimate the probability of hospital admission of these patients with maintained spontaneous circulation. Initial rhythm was the most important predictor of prehospital survival. Patients with shockable initial rhythm, lower age, lower first measured heart rate and higher first measured blood pressure had a better chance of hospital admission with spontaneous circulation. ECG localization of STEMI was nonsignificant in multivariable logistic regression, but the inclusion of this variable in the model improved the model fit.

### Comparison of previous studies and clinical implications

The previously published observation that OHCA was less likely to occur with increasing age (OR = 0.93 [95% CI: 0.89–0.97 for + 10 years], *p* < 0.001) among patients who were successfully admitted to the hospital) can be countered by our results. These results showed that older patients with resuscitated STEMI had significantly higher prehospital mortality (OR = 1.036 for years [95% CI = 1.02–1.05], *p* < 0.001), thus, a smaller proportion of older patients with resuscitated STEMI were successfully transferred to hospital and thus were not included in hospital databases [12]. 

Age, heart rate and systolic blood pressure are already known as predictors of STEMI mortality, based on widely validated scores developed from hospital-databases [13, 14]. In these algorithms, cardiac arrest is also predictive of mortality; however, it has not been analyzed separately based on initial rhythm. In a recent hospital-data study, Nagy et al. reported that initial rhythm may also predict longer-term mortality in resuscitated patients with STEMI, however, heart rate was shown to be a more significant predictor of 30-day mortality [15]. 

In our present study, the probability of mortality in patients with cardiac arrest due to a non-shockable rhythm during prehospital care was exceptionally high. There is also the possibility that a proportion of patients might have pseudo-PEA (pseudo-pulseless electric activity) instead of true cardiac arrest. In addition, the poor pulse detection ability of healthcare providers should also be considered [16–18]. 

According to some publications, the advanced life support (ALS) algorithm may not be the most effective therapy for patients with pseudo-PEA. Prosen et al. revealed that echocardiographic verification of pseudo-PEA state in out-of-hospital cardiac arrest allowed additional vasopressor treatment and cessation of chest compressions, and was associated with significantly higher rates of RoSC, survival to discharge and good neurological outcomes [19]. 

Breitkreuz reported that echocardiography at peri-resuscitation setting may play a role in determining whether a patient has “pseudo-PEA” (coordinated electrical activity, no palpable pulse, but coordinated cardiac activity) or “true-PEA” (electrical activity but no detectable cardiac motion and no palpable pulse, electromechanical dissociation [EMD]). Thus, EMD confers a poor prognosis with only 8% of patients surviving to hospital admission. In contrast, pseudo-PEA confers a better prognosis, with 55% of patients surviving to hospital admission. Thus, the echocardiographic distinction may prove relevant in the future for prognosis, optimization, and more tailored administration of peri-resuscitation drug therapy [20]. 

Aichinger and Zechner et al. reported, that cardiac standstill at any time during CPR had a positive predictive value of 97.1% for death at the scene; therefore, these results support the idea of focused echocardiography as an additional criterion in the evaluation of outcomes in CPR patients and demonstrate its feasibility in the prehospital setting [21]. 

In summary, initial rhythm, age, first measured heart rate and blood pressure, and the ECG localization of STEMI can be used to estimate the probability of hospital admission with maintained spontaneous circulation after OHCA-CPR in patients who were diagnosed with STEMI even before cardiac arrest. Initial rhythm was the most important predictor of prehospital survival, as patients with an initial non-shockable rhythm were at an extremely high probability of mortality. An unknown proportion may have included those who did not even have true cardiac arrest, but severe circulatory failure without palpable pulse (pseudo-PEA). As point-of-care ultrasound examinations are becoming more widely available, and some studies indicate that ACLS (advanced cardiovascular life support) may not be the best therapy for pseudo-PEA patients, further studies are needed to compare prehospital diagnostic and therapeutic alternatives for these patients to improve the possibility for definitive treatment in hospital and increase their chances of survival.

## Data Availability

The data that support the findings of this study are available from Hungarian National Ambulance Service but restrictions apply to the availability of these data, which were used under license for the current study, and so are not publicly available. Data are however available from the authors upon reasonable request and with permission of Hungarian National Ambulance Service.

## Abbreviations

ACLS: Advanced cardiovascular life support
AIC: Akaike’s Information Criterion
ALS: Advanced life support
CI: Confidence interval
CPR: Cardiopulmonary resuscitation
df: Degrees of freedom
ECG: Electrocardiogram
etCO2: End-tidal carbon dioxide
ICD (codes): International Classification of Diseases
IQR: Interquartile range
LBBB: Left bundle branch block
min: Minute(s)
mmHg: Millimeters of mercury
OHCA: Out-of-hospital cardiac arrest
OR: Odds ratio
PEA: Pulseless electric activity
RBBB: Right bundle branch block
RoSC: Return of spontaneous circulation
SE: Standard error
STEMI: ST-elevation myocardial infarction
